# Keeping staff members safe

**Published:** 2021-07-20

**Authors:** Astrid Leck

**Affiliations:** 1 Assistant Professor and Mcrobiologist: London School of Hygiene & Tropical Medicine, London, UK.

**Without staff members, we do not have a health care service. Keeping staff members safe is therefore of utmost importance if we want to provide safe eye care to our patients.**

**Figure:**
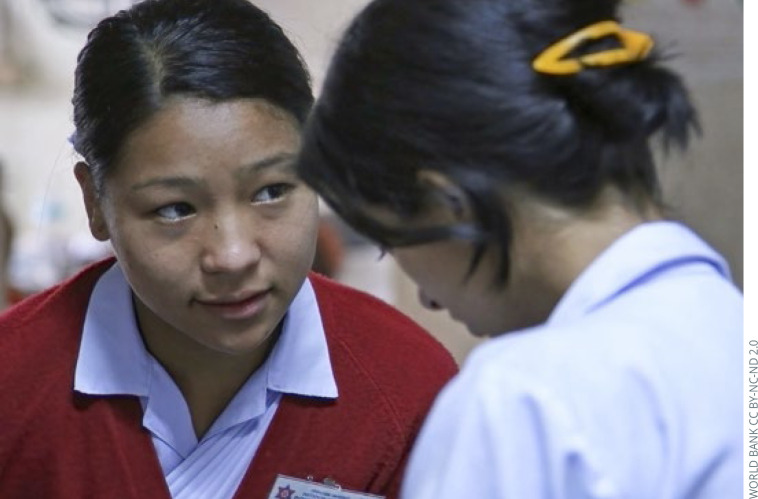
By taking time to listen to one another’s concerns, colleagues can be an important source of support. **NEPAL**

On World Patient Safety Day 2020, the World Health Organization (WHO) reminded governments that they have a legal and moral responsibility to ensure the health, safety and wellbeing of health workers and announced a Health Safety Charter (**bit.ly/safeWHO**),^1^ which calls on member states to:

1. **Establish synergies between health worker safety and patient safety policies and strategies**, including training programmes for all health workers and incident reporting systems.
2. **Develop and implement national programmes for occupational health and safety of health workers** to ensure that all health workers have regulatory protection of their health and safety at work.
3. **Protect health workers from violence in the workplace**. Violence in the workplace manifests as inequality, abuse, harassment, discrimination, stigmatisation and conflict in health care settings. Any form of violence against health workers is unacceptable.
4. **Improve mental health and the psychological well-being of health workers.** Many health workers operate in high-demand, high-risk and high-stress work settings for long hours.
5. **Protect health workers from physical and biological hazards**. Health workers face multiple physical, biological and ergonomic hazards, including exposure to infections, sharps, falls, radiation, chemicals, fire and electrical hazards, or musculoskeletal disorders due to poor ergonomics in handling patients and lifting heavy equipment.

**“It is the responsibility of the eye hospital or clinic, as the employer, to provide personal protective equipment (PPE) for staff members.”**

The importance of staff health cannot be overemphasised. Health and safety risks to health workers can lead to risks for patients, including patient harm and adverse patient outcomes. Commenting on the role of health workers during the COVID-19 pandemic, WHO Director-General Tedros Adhanom Ghebreyesus said: “No country, hospital or clinic can keep its patients safe unless it keeps its health workers safe.” In this article we consider how this can be done at the facility or hospital level.

Clinical and non-clinical members of staff have daily contact with patients and/or infectious material, and health care workers are considered to be at significant risk of acquiring or transmitting hepatitis B, influenza, measles, mumps, rubella, and varicella. Immunisation of all staff members protects them against these diseases, and also prevents staff members from infecting patients. Vaccination is therefore an essential part of infection prevention and control programmes.^2^

It is the responsibility of the eye hospital or clinic, as the employer, to provide personal protective equipment (PPE) for staff members whenever there is a risk to their health or safety which cannot be controlled by other means. Always ensure that there is enough PPE available, that it fits well, and that the type and quality is suitable for the work being done.

Staff members may have underlying health conditions for which adaptations must be made. For example, health workers who are pregnant must be protected from hazards and risks in the workplace and offered suitable alternative work if it is not possible to carry out their normal duties safely.

## Injury prevention

Injury within the workplace can take many forms. Prevention of sharps injuries (including needle-stick injuries) is a specific priority announced at World Patient Safety Day in 2020. Because of the risk of infection, these injuries can cause significant worry and stress, so make sure health workers have access to post-exposure prophylaxis as per the local clinical guidelines, as well as testing, advice, and counselling.

Another significant cause of injury in health care settings is associated with manual handling, i.e., lifting and moving patients, equipment, laundry, supplies, and waste. Neck and back pain are common among eye care workers due to the awkward angles and positions required during eye examination and surgery. It is important to minimise your risk of these occupational injuries in the long-term. You may need to adjust your position, your equipment, and the patient’s position to improve comfort. Where feasible, use ergonomically designed ophthalmic equipment and furniture to prevent eye injury, repetitive strain injury, and musculoskeletal injury. Equipment should only be used if it is functioning optimally and well maintained. In the office environment, keep all screens at eye level when seated.

## A safe environment

All clinical facilities need to prioritise a safe environment, including:

- Access to clean water, sanitation, and hygiene
- Disinfection of the clinical environment
- Provision and maintenance of ventilation systems to maintain good air flow and a comfortable temperature within the workplace
- Electrical safety
- Reduction of excessive noise levels which could result in hearing damage
- Prevention of exposure to harmful radiation, such as X-rays.

It is important to ensure that services provided by external contractors are adequately monitored and supervised.

## Protecting health workers from violence

Staff security is also an important issue, especially when responsibilities require lone working, as is the case for night shift workers and community health workers visiting patients in their homes. Consider how staff members can travel to and from home safely at night, as road traffic accidents may be more frequent during this period. It is advisable to provide safe transport where security is a concern.

Violence at work, including bullying and harassment, should not be tolerated or ignored. It is important to create policies and strategies to prevent and effectively manage concerns and events relating to any of these issues, for example, by setting up a supportive and confidential reporting system.

Aggression towards staff members by patients and members of the public should not be accepted. Set out standards of conduct for staff members as well as patients, relatives, and visitors to the hospital or clinic. Promote a culture of zero tolerance towards violence or aggression against health care workers, for example, by using posters to convey this message.

**Figure:**
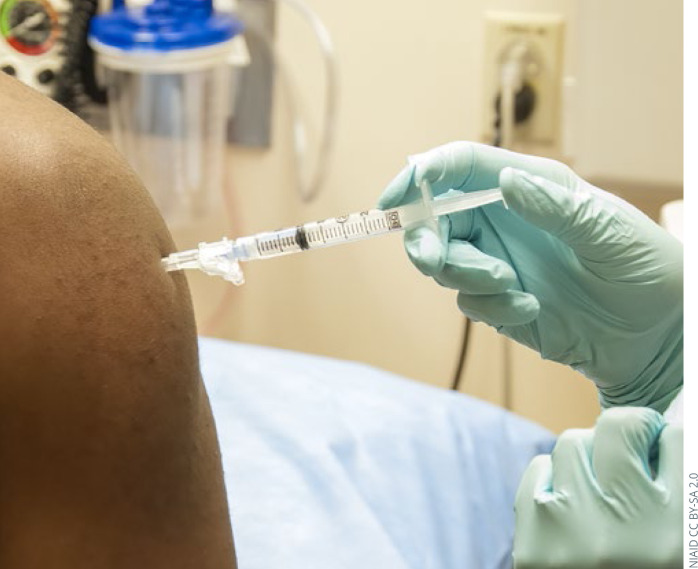
An important way to keep staff members safe is to offer recommended immunisations. **USA**

## Improving mental health and the psychological wellbeing of health workers

Work-related stress and burnout are ongoing challenges for health care workers; this has been intensified by the additional pressures of caring for those affected by the COVID-19 pandemic and the disruption it causes to routine patient services. There are several things you can do to mitigate this.

1. Consider how to encourage and support staff members. Be aware that staff members may face all sort of stresses outside the workplace; reassure them that this is normal and that they are supported. It may be helpful to offer staff members access to counselling or other forms of psychological support.
2. Optimise staff scheduling. Plan the length of shifts and the composition of the eye team to optimise workload and task sharing so staff members can take regular rest breaks and have time off work.
3. Establish a culture of learning instead of blaming. Develop a process whereby all staff members can confidentially report adverse safety events or near misses without fear of repercussions.
4. Set up a confidential reporting system where staff members can report any form of bullying or harassment that they experience in the work place.

## Setting up good systems for safety

Good safety systems, backed up by documentation, play a vital role in creating a safer workplace for staff members and patients. Strive to adopt international and national occupational health and safety standards and keep up to date with local legislation.

It is the responsibility of health care management to create documentation for all clinical and non-clinical activities within the eye care setting that can have an impact on patient or staff member safety. These include risk assessments, standard operating procedures, inspection reports, and maintenance records.

The aim of **risk assessments** is to eliminate, reduce or control the risk to patients and staff members associated with a procedure or activity. **Standard operating procedures (SOPs)** document safe systems of work.

As new infectious diseases emerge, SOPs must be revised to look for activities that can pose a risk to patients and health workers; for example, vision testing, refraction, slit lamp examination, and fundoscopy, to name just a few examples. SOPs should be reviewed and audited regularly.

Management is also responsible for creating robust systems that are well documented (e.g., posters in the staff room or in corridors) so that everyone knows how to respond to situations such as:

- Needle-stick injuries
- Accidental spillage, and harmful exposure to, chemicals
- Health care associated infections
- Near-misses.

Injury and incident reporting is time sensitive and often requires immediate action, so staff members must know where and how to report these. It is also the responsibility of management to ensure there is mechanism in place for prevention (e.g., availability of safety boxes for disposal of sharps in every section of the facility) and compensation in case injuries occur, as per the local guidelines.

## Training

It is important to offer regular training for staff members on how they can keep themselves safe. Take care to train all staff members, not just health care workers. For example, cleaning personnel and external contractors also need to be familiar with how infection spreads, how they may need to protect themselves, and what they should report. Recruiting staff champions to model good practice is a recognised approach to encourage uptake and compliance.
